# Barriers to recruiting and retaining psychosis carers: a case study on the lessons learned from the Caring for Caregivers (C4C) trial

**DOI:** 10.1186/s13104-019-4832-9

**Published:** 2019-12-17

**Authors:** Cassie M. Hazell, Christina J. Jones, Aparajita Pandey, Helen E. Smith

**Affiliations:** 1 School of Social Sciences, University of Westminster, London, W1W 6UW UK; 2 University of Surrey, Surrey, Guildford, GU2 7XH UK; 3 Sussex Partnership NHS Foundation Trust, Research and Development, Hove, BN3 7HZ UK; 4 Lee Kong Chian School of Medicine, Nanyang Technological University Singapore, Singapore, 308232 Singapore; 5 Division of Public Health and Primary Care, Brighton and Sussex Medical School, Falmer, Brighton, BN1 9PH UK

**Keywords:** Recruitment, Retention, Barriers, Trial, Carer, Psychotic disorders

## Abstract

**Objective:**

Carers play an important role within the UK mental health system. Those carers who support persons with psychosis can experience a reduction in their own physical and mental health. As part of the Caring for Caregivers (C4C) trial, we piloted a writing intervention (Positive Written Disclosure) that has been shown to improve wellbeing in other populations. Although we reached our recruitment target, we encountered several barriers that made recruitment slower than anticipated. This paper synthesises the process data collected during the C4C trial that relates to the barriers to recruiting and retaining psychosis carers.

**Results:**

We encountered four main carer-specific barriers to the recruitment and retention of participants in our study. These were: (1) poor relationship with mental health clinicians, (2) conflicting with the care recipient’s (CR) needs, (3) lack of spare time, and (4) lack of services for mental health carers. The interventions to assist carers need to be informed by robust evidence and this requires trials that reach their recruitment targets. By sharing our practical experiences other researchers and clinicians can modify their practices to minimise recruitment difficulties and delay.

*Trial registration* ISRCTN79116352. Retrospectively registered (before the final participant was recruited) on 23rd January 2017

## Introduction

Difficulty recruiting participants for research is not uncommon. Almost half of clinical trials fail to achieve their original recruitment targets in the specified time [1]. Failure to reach recruitment targets can weaken the conclusions generated from the trial. Furthermore, there is evidence suggesting that recruiting carers may be particularly challenging [2, 3]. Our Caring for Caregivers (C4C) trial of Positive Written Disclosure (a self-directed, time-limited writing therapy) required us to recruit 60 older adults caring for someone with psychosis [4]. Throughout the trial recruitment was difficult. We had originally planned to recruit sufficient numbers of carers from a single site, but in reality we had to extend the recruitment period and add three additional sites to reach our recruitment target.

This paper offers an explanatory case study outlining the challenges to recruiting carers to a trial and ways to minimise these. This case study merges our learning, based on trial process data, with the existing literature on carers. By sharing our learning, other researchers can consider the potential barriers and proactively incorporate potential solutions within their study design.

This case study paper aims to identify: (1) the barriers to recruiting and retaining psychosis carers; and (2) practical ways to overcome these barriers.

## Main text

### Method

We did not anticipate recruitment difficulties, and therefore did not plan a proactive formal evaluation of recruitment methods. Instead, we offer a case study using the process data collected during the C4C trial. CH and AP reviewed the recruitment databases, contact logs, and the minutes from the research team, trial steering committee (TSC) and lived experience advisory panel (LEAP) meetings. They extracted the recruitment conversion rates (how many referrals went on to provide consent), reasons why participants declined to be contacted or consent (recruitment databases and contact logs), and examples of good practice where we had managed to successfully recruit participants (recruitment databases and meeting minutes). Finally, the data and its interpretation were discussed with the core research team and our LEAP.

### Results

We utilised a range of recruitment methods to reach our target, including promoting the study via community and inpatient mental health services, GPs, mental health and carer third sector organisations, recovery colleges, social media, and posters in community buildings (e.g. libraries, community centres)—full recruitment details can be found in the study protocol [4]. Of the 408 referrals we received, 63 (15%) carers consented to participate in the trial. In this paper we are able to share what we have learnt from the 345 cases where people were referred but did not go on to participate. We have identified four barriers to recruiting psychosis carers (Table 1).

**Table 1:** Summary of the four main barriers to recruiting psychosis carers

Barrier	Summary	Suggested approach to minimise impact
Poor relationships with mental health clinicians	A negative relationship with the referring clinician is likely to make the carer less receptive to the research study	Target those services that are carer friendly
Conflicting with the care recipient’s needs	If the care recipient does not approve of the study then it can make it difficult for the carer to be involved	Be transparent about the details of the project with any care recipients that make contact, while protecting participant confidentiality Make study documentation available to the care recipient Use terms such as ‘family and friends’ instead of carer
Lack of spare time		
Carers’ frustration at the delay between research and implementation	Research takes time, but carers are looking for immediate solutions to their problems	Keep carers updated about the progress of the study Develop a clear and realistic dissemination and implementation plan, ideally with input from a lived experience group
Impact of caregiving demands	It can be difficult to balance the time commitment involved in caring and being a research participant	Make the study design flexible and pragmatic to accommodate for the demands of caring Minimise the burden of the research on participants, for example keeping questionnaires short Allow for generous attrition rates (i.e. > 33%) in sample size calculations
Lack of services for mental health carers	Carers can be hard to reach as the usual means of advertising clinical trials are not available	Be creative in how you promote the research study, beyond the strategies usually employed in clinical trials Consider making relationships with and promoting your project in settings beyond healthcare services Encourage snowball recruitment

#### Barriers

1. Poor relationships with mental health clinicians:
  Our initial plan was to recruit carers via mental health services, asking clinicians to pass on the C4C study information to the carers of their psychosis patients. Unfortunately, this strategy proved to be largely ineffective as all the participants who actively declined to participate in the trial (14; 4%) came via mental health clinicians. Many carers expressed their frustrations with mental health staff, and perceived discussion of the C4C study was being used as way of evading and deflecting enquiries of what help was available for the person they cared for.
2. Conflicting with the care recipient’s (CR) needs:
  Although the C4C study did not recruit CRs, their influence was very much felt throughout the study. Carer participation in this project was frequently not supported by CRs for two reasons: unacceptability of the negative connotations of CR; and because some carers feared their CR would become suspicious, or paranoid, about the research, and wished not to risk increased distress.
3. Lack of spare time:
  Carers’ time is precious, so decisions to participate in a research study will be carefully considered. Shortage of time was contributory to two sub-barriers: (i) carers’ frustration at the delay between research and implementation, and the (ii) impact of caregiving demands.
  i. Carers’ frustration at the delay between research and implementation:
    Some carers expressed their frustration at the time it takes for research findings to be implemented in healthcare. Several people who were invited to participate in our study mentioned their previous research engagement and their disappointment that they “never found out what happened in the end”. Furthermore, carers were surprised when they found out the C4C study would take more than 2 years. Understandably, if they had a CR who was currently in “crisis”, their priority was to support their CR. Taking a longer-term perspective and addressing their own needs could not compete.
  ii. Impact of caregiving demands:
    Changes in the intensity of the caring role can happen quickly. The C4C study assessed carers over 6 months. Only two carers formally withdrew from the trial; both of whom had initial capacity to participate but later were struggling with their CR’s “relapse”. Other carers cited CR relapse as a reason for not completing assessments on time: in our trial, 6 (10%) participants required assessment packs to be re-sent for at least one of the time-points.
4. Lack of services for mental health carers:
  All the barriers described above occurred after the researchers had met with psychosis carers, creating the initial encounter proved even more challenging. Carers are a hard to reach population, with a limited number of carer organisations who could act as allies in promoting research. We attended four generic carer events but met only one carer who was eligible for the study. However, where mental health carers’ groups were available, they were helpful. Unexpectedly, our attempts to recruit via GPs returned very small numbers because of the incompleteness of their carer registers, and that even where registers existed they did not systematically record the condition of the CR. We sent 114 study invitations to people who identified as ‘carers’ from 10 GPs; only 7 (6%) of these consented to participate in the trial. The disparity between the referral and consent rates could reflect a lack of interest, but is more likely because only a small percentage were providing care to someone with psychosis and therefore eligible.

#### Strategies

1. Poor relationships with mental health clinicians:
  To overcome this barrier requires mental health services to mend their relationships with carers. In the longer term this barrier may be diminished with the popularisation of initiatives such as the Triangle of Care [5] and the friends and family policies of Early Intervention in Psychosis (EIP) services [6]. In the interim, we recommend that researchers target recruitment efforts on those services that have a tradition of involving carers, as indicated by the appointment of Carer Leads or Carer Liaison Officers.
2. Conflicting with the care recipient (CR) needs:
  In attempt to reduce these barriers, we adopted a position of openness and transparency, making explicit reference to our definition of the term carer on all participant-facing documentation (“any person who provides unpaid support to a partner, child, relative or friend who couldn’t manage to live independently or whose health or wellbeing would deteriorate without this help” [7]). All of the study documents were freely available online. On reflection we could have also developed study materials specifically for CRs too and offered CRs the opportunity to contact the research team (whilst of course upholding participant confidentiality). Additionally, we could have replaced the term ‘carer’ with less personally threatening terms such as ‘friends and family’. In doing so we would need to have acknowledged that being related to someone with psychosis does not automatically qualify the person as a carer; the trial inclusion/exclusion criteria would need to be crafted carefully to ensure the caring role was being undertaken.
3. Carers’ frustration at the delay between research and implementation:
  The needs of carers’ require attention at the research design stage. Carers are more likely to become involved in studies that they “believe in”; carers appreciate research with a clear pathway to completion and studies that will bring about tangible changes. Researchers must resist any temptation to oversell their projects to boost recruitment; failing to deliver on promises of relevance and impact, leaves carers feeling exploited [8] and the likelihood of participating in another research study in the future is threatened.
  When asked, all our sample requested copies of the study results, and even where carers were ineligible, they expressed an interest in hearing about the findings. Therefore, in our dissemination plan we demonstrated a willingness to share our findings as widely as possible, including dissemination via statutory and third sector organisations. The dissemination plan was summarised in the participant information sheet and we were always explicit that as a feasibility study further research would be needed before implementation. Regular project newsletters updated participants with study progress, changes to the protocol or study timeline, and reminded those participants who had completed their data collection that the study was still ongoing.
4. Impact of caregiving demands:
  Attrition and delayed follow up assessments are unavoidable when working with carers and so wherever possible the research design should offer flexibility in processes and procedures. To facilitate involvement, we offered carers a choice in the method of assessment (in-person, phone, online, post), and we minimised travel time and costs by seeing participants in their homes. Carers took advantage of this flexible approach with 5 (8%) participants electing to complete at least one assessment online instead of via post, and all but one carer electing to complete the exit interview over the phone instead of in-person. Overall, our rates of retention were good (97%) as was data completion (84% at 6 months), demonstrating that even busy carers can be retained if you do not ask too much from them.
5. Lack of services for mental health carers:
  With the inadequacy of carers’ registers, we were largely reliant on GPs’ knowledge of their caseload. An alternative approach suggested by Sampson et al. [9] is to identify carers by searching patient records to find those cohabiting with a person experiencing the condition of interest. This approach assumes that the person living with someone with a health problem will be the person providing care, but as discussed previously this may not always be the case. For the C4C project this resource intensive approach to recruitment would have had limited utility as many people with psychosis tend to live in supported accommodation [10, 11]. Beyond conventional recruitment strategies, we would encourage researchers to be creative. We promoted the C4C study in a variety of locations that are not traditionally used for clinical trials, including community centres, libraries, pharmacies, recovery colleges, adult education organisations, emancipatory mental health groups and local authority premises. We also used snowball sampling, encouraging participants to recommend the research to other carers they knew.

### Discussion

#### Barriers

The majority of trials recruiting carers of people with psychosis do so solely via mental health services (e.g. [12–14]). The quality of the recruitment processes used in such trials has improved in recent years but, according to trial quality assessment criteria, remains below standard [15]. There is little published looking at the barriers to recruiting carers of people with psychosis, but of the few studies that exist there are some emerging patterns. For example, when recruiting siblings of Early Intervention in Psychosis Services (EIS) service-users, the researchers found recruitment was impeded by difficulties making contact with carers, problematic relationships between clinicians and carers, and the care recipients’ concerns about the research [16]. Motivated by the consistency of these two studies, we would encourage researchers to be attentive to our learning.

#### Strategies

We have identified specific strategies to overcome each of the barriers identified. However, our foremost recommendation is for researchers to integrate themselves with the local carer community by attending carer events, supporting and promoting third-sector organisations wherever possible, and becoming attuned to the day-to-day difficulties faced by carers in their locality. Dedicating time to such activities will help the research team to build relationships and make contacts willing to go the ‘extra mile’ to support your study. Well-resourced public and patient involvement (PPI) is another way that researchers can start to get involved in the carers’ community. This has the additional benefit of enhancing the relevance and impact of the research [17]. Our PPI consultants helped us to make sense of recruitment barriers as they arose and many of the suggestions we offer came from conversations with them.

### Conclusion

Research involving carers of people with psychosis is much-needed but challenging. There are a number of barriers to recruiting carers for research, but we hope that sharing our experiences from the C4C trial will enable other researchers to assess the risk of these barriers in the context of their own studies, and plan accordingly. Proactive consideration of the potential barriers to recruitment at the research design stage will increase the likelihood of trials reaching their targets on time.

## Limitations

- The findings only reflect the carers who were willing to share their reasons for not taking part.
- The study used process data rather than primary research data.
- The study is observational, and therefore we do not have comparative data to formally evaluate each recruitment method.
- Our results are specific to older adult carers of people with psychosis, and it is therefore possible that different and/or additional barriers may be present amongst younger carers.

## Data Availability

The datasets generated and/or analysed during the current study are not publicly available as this paper reports on process data for which we do not have permission to share publicly; but some data are available from the corresponding author on reasonable request.

## Abbreviations

C4C: Caring for Caregivers trial
PWD: Positive Written Disclosure
EIP: early intervention in psychosis
CR: care recipients
PPI: public and patient involvement
NHS: national health service
GP: general practice
LEAP: lived experience advisory forum
